# Association of anthropometric measures across the life-course with refractive error and ocular biometry at age 15 years

**DOI:** 10.1186/s12886-020-01480-3

**Published:** 2020-07-08

**Authors:** Alison Bruce, Neema Ghorbani Mojarrad, Gillian Santorelli

**Affiliations:** 1 Bradford Institute for Health Research, Bradford Teaching Hospitals NHS Trust, Duckworth Lane, Bradford, BD9 6RJ England; 2 Department of Optometry and Vision Sciences, University of Bradford, Bradford, BD7 1NX England

**Keywords:** Refractive error, Ocular biometry, Myopia, Height, Weight, Life-course, Maternal, Birth, Teenage, ALSPAC

## Abstract

**Background:**

A recent Genome-wide association meta-analysis (GWAS) of refractive error reported shared genetics with anthropometric traits such as height, BMI and obesity. To explore a potential relationship with refractive error and ocular structure we performed a life-course analysis including both maternal and child characteristics using data from the Avon Longitudinal Study of Parents and Children cohort.

**Methods:**

Measures collected across the life-course were analysed to explore the association of height, weight, and BMI with refractive error and ocular biometric measures at age 15 years from 1613children. The outcome measures were the mean spherical equivalent (MSE) of refractive error (dioptres), axial length (AXL; mm), and radius of corneal curvature (RCC; mm). Potential confounding variables; maternal age at conception, maternal education level, parental socio-economic status, gestational age, breast-feeding, and gender were adjusted for within each multi-variable model.

**Results:**

Maternal height was positively associated with teenage AXL (0.010 mm; 95% CI: 0.003, 0.017) and RCC (0.005 mm; 95% CI: 0.003, 0.007), increased maternal weight was positively associated with AXL (0.004 mm; 95% CI: 0.0001, 0.008). Birth length was associated with an increase in teenage AXL (0.067 mm; 95% CI: 0.032, 0.10) and flatter RCC (0.023 mm; 95% CI: 0.013, 0.034) and increasing birth weight was associated with flatter RCC (0.005 mm; 95% CI: 0.0003, 0.009). An increase in teenage height was associated with a lower MSE (− 0.007 D; 95% CI: − 0.013, − 0.001), an increase in AXL (0.021 mm; 95% CI: 0.015, 0.028) and flatter RCC (0.008 mm; 95% CI: 0.006, 0.010). Weight at 15 years was associated with an increase in AXL (0.005 mm; 95% CI: 0.001, 0.009).

**Conclusions:**

At each life stage (pre-natal, birth, and teenage) height and weight, but not BMI, demonstrate an association with AXL and RCC measured at age 15 years. However, the negative association between refractive error and an increase in height was only present at the teenage life stage. Further research into the growth pattern of ocular structures and the development of refractive error over the life-course is required, particularly at the time of puberty.

## Background

The influence of prenatal factors in the development of conditions such as heart disease, diabetes, and obesity is well reported, with maternal health and the prenatal environment known to influence development throughout the life-course [1]. Early life influences on myopia have also been explored, with childhood growth between birth and ten years showing some effect [2, 3]. Childhood growth is known to be associated with maternal metabolism [4] and there is evidence that maternal stature influences the development of myopia, with maternal height being associated with myopia in adulthood [5]. Refractive error is determined by the ocular structure of the eye with the key components being axial length, corneal power and lens power; myopia occurs when axial length exceeds the focal length [6]. Axial length is highly correlated with refractive error and increased axial length related to myopia is well documented [3, 6, 7]. A recent Genome-wide association meta-analysis (GWAS) of refractive error investigated the overlap of genes identified in refractive error with other common traits and reported shared genetics with anthropometric traits such as height, BMI and obesity [8]. In order to explore a potential relationship between stature, refractive error and ocular structure we performed an analysis including both maternal and child characteristics across the life-course using data from the Avon Longitudinal Study of Parents and Children (ALSPAC) cohort [9, 10].

## Methods

### Study population

ALSPAC is a birth cohort of mothers and their children born between 1991 and 1992 residing in the county of Avon, South West England. A variety of measures have been collected prospectively from the participants, with the methodology of collection of ALSPAC measures detailed previously elsewhere [9, 10].

The ALSPAC study website contains details of all the data that is available through a fully searchable data dictionary and variable search tool. (https://www.bristol.ac.uk/alspac/researchers/our-data/).

**Fig. 1:**
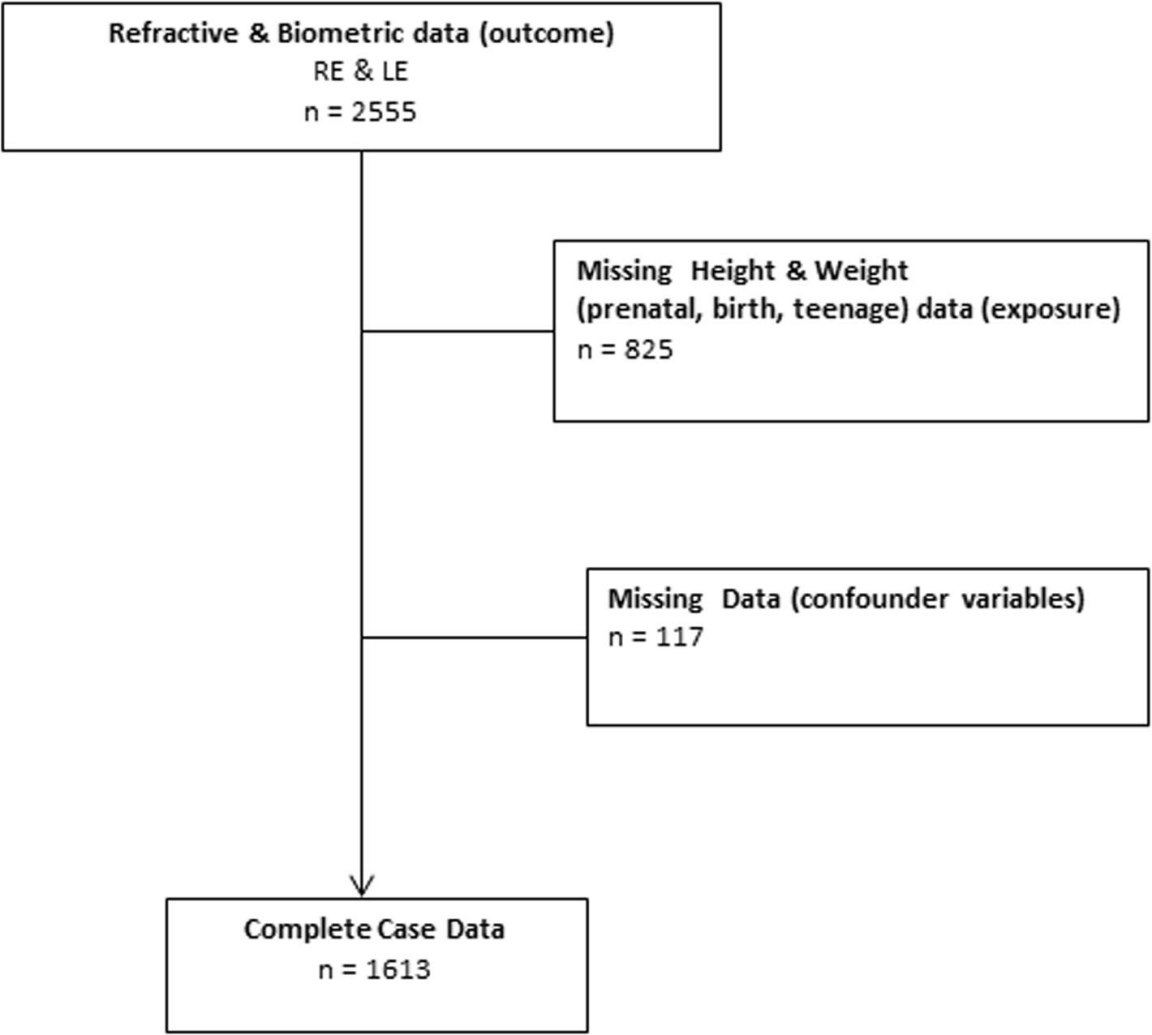
Flow chart describing the data of the participants included in the analysis

Refractive and biometric data were available for 2555 children. We excluded individuals with missing exposure (*n* = 825) and covariable (*n* = 117) data; the final cohort for this study is 1613 (Fig. 1).

### Outcome measures

The three main outcome measures were the mean spherical equivalent (MSE) of refractive error (dioptres), axial length (AXL; mm), and radius of corneal curvature (RCC; mm). All outcome measures were collected from the participants at age 15 years. Non-cycloplegic autorefraction was used to estimate the refractive error (Canon R50 instrument, Canon USA Inc., Lake Success, NY). This was measured in both eyes and then used to calculate the MSE (sphere plus half of cylinder). AXL and RCC were measured using Zeiss IOL master (Carl Zeiss Meditec, Welwyn Garden City, UK). The mean measure of both eyes for each outcome measure was used in the analyses, coded as continuous variables.

### Exposure variables

We examined variables previously identified as potentially being associated with myopia: height [5, 8], weight [8] and BMI [8] were examined at three life stages: the prenatal period (maternal factors), birth, and at 15 years of age. Maternal height (cm) [5], pre-natal weight (kg) [8], and BMI (kg/cm2) [8], were self-reported by the mother and collected by questionnaire. Birth weight [3, 11] was extracted from medical records and birth length [8] was measured by the ALSPAC team after birth (median 1 day, range 1–14 days) using a Harpenden neonatometer. Teenage height (measured using Harpenden Stadiometer) and weight (Tanita Body Fat Analyser Model TBF 305; Tanita Europe Ltd., Amsterdam, The Netherlands) were collected at 15 years of age at research clinic attendances.

### Covariables

Potential confounding variables previously reported to be associated with myopia throughout the life course were adjusted for within each life stage model: Maternal age at conception (years) [5], maternal education level [5], and parental socio-economic status [5] were self-reported by the mother and collected by questionnaire. Gestational age [3, 11], breast-feeding [5] and gender [7] were extracted from medical records.

### Statistical analyses

All statistical analyses were performed using Stata (v 15). Initially, univariable linear regression was performed to examine the relationship between each exposure variable and each covariable (listed above) with each of the three outcome measures (MSE, AXL and RCC) at each life stage; those with the strongest associations (*p* < 0.20) were included in the multivariable linear regression models. In each multi-variable model interactions between gender and height and gender and weight were included, retaining those that were significant in the final models.

## Results

Participant characteristics are summarised in Table 1. In the univariable analysis, BMI was not found to be associated with any of the three outcome measures therefore we examined the associations of height and weight, but not BMI, in each of the multivariable analyses. Univariate analysis found females had a reduced teenage AXL (− 0.499 mm; 95% CI: − 0.583,-0.415) and steeper corneas (RCC) (− 0.100 mm; 95% CI: − 0.125,-0.075) compared to males, however no interactions were identified.

**Table 1:** Characteristics of participants (mothers and children)

Mother	
Age (years)	29.37 (4.4)
Height (cm)	164.24 (6.4)
Weight (kg)	62.19 (10.8)
Mother’s education	
CCE/vocational	272 (16.9)
O level	570 (35.3)
A-level	466 (28.9)
Degree equivalent	305 (18.9)
Social Class*	
I	300 (18.6)
II	526 (32.2)
III non-manual	606 (37.6)
III manual	77 (4.8)
IV/V	104 (6.5)
**Child**	
Gender	
Male	748 (46.4%)
Female	865 (53.6%)
Gestational age at birth (weeks)	39.60 (1.6)
Birth weight (g)	3445.05 (508.5)
Breast Fed	
No	212 (13.1%)
Yes	1401 (86.9%)
Myopia at 15 years (average MSE ≤ 0.50 D)	
No	1355 (84.0%)
Yes	258 (16.0%)

Values are numbers (%) or mean (SD)

Family social class* is defined as the highest household reported occupation, ranging from I (professional), II,III (subdivided manual and non-manual) IV, and V

### Life stage: prenatal (Table 2)

The factors found to be significant in univariable analysis and included in the multivariable prenatal life stage model were: maternal age at conception (years), maternal height (cm), pre-natal weight (kg), maternal education level, and parental socio-economic status.

**Table 2:** Associations between prenatal (maternal) factors and refractive error mean spherical equivalent (MSE in dioptres), axial length (AXL in mm) and radius of corneal curvature (RCC in mm) measured at age 15 years

	Multivariable analysis	***P*** value
**Mean Spherical Equivalent**	**(ß coefficient (95% CI))**	
Maternal weight (kg)	0.001 (− 0.003,0.005)	*p* = 0.530
Maternal height (cm)	0.0002 (−0.006,0.006)	*p* = 0.050
**Axial length**		
Maternal weight (kg)	0.004 (0.0001,0.008)	*p* = 0.042
Maternal height (cm)	0.010 (0.003,0.017)	*p* = 0.006
**Corneal Curvature**		
Maternal weight (kg)	0.0004 (−0.0009,0.002)	*P* = 0.553
Maternal height (cm)	0.005 (0.003,0.007)	*p* < 0.001

No prenatal association was found between maternal height or weight with MSE. Maternal height was positively associated with a longer teenage AXL (0.010 mm; 95% CI: 0.003, 0.017) and greater RCC (0.005 mm; 95% CI: 0.003, 0.007), increased maternal weight was also associated with an increase in AXL (0.004 mm; 95% CI: 0.0001, 0.008) (Table 2).

### Life stage: birth (Table 3)

The multivariable model of the birth life stage included gestational age (weeks), birth weight (g), and length at birth (cm). No association was found between birth length or weight with MSE. Birth length was associated with an increase in teenage AXL (0.067 mm; 95% CI: 0.032, 0.10) and flatter RCC (0.023 mm; 95% CI: 0.013, 0.034) respectively. An increase in birth weight was also associated with flatter RCC (0.005 mm; 95% CI: 0.0003, 0.009).

**Table 3:** Associations between birth factors and refractive error mean spherical equivalent (MSE dioptres), axial length (AXL mm) and radius of corneal curvature (RCC mm) measured at age 15 years

	Multivariable analysis	***P*** value
**Mean Spherical Equivalent**	**(ß coefficient (95% CI))**	
Birth weight (per 100 g)	0.002 (− 0.011,0.014)	*p* = 0.793
Birth length (cm)	−0.025 (− 0.056,0.007)	*p* = 0.125
**Axial length**		
Birth weight (per 100 g)	0.006 (−0.008,0.021)	*p* = 0.381
Birth length (cm)	0.067 (0.032,0.102)	p < 0.001
**Corneal Curvature**		
Birth weight (per 100 g)	0.005 (0.0003,0.009)	*p* = 0.035
Birth length (cm)	0.023 (0.013,0.034)	p < 0.001

### Life stage: teenage (15 years) (Table 4)

Factors included in the teenage life stage model included weight (kg) and height (cm) at 15 years of age. An increase in teenage height was associated with a reduction in MSE (− 0.007 D; 95% CI: − 0.013, − 0.001), an increase in AXL (0.021 mm; 95% CI: 0.015, 0.028) and flatter RCC (0.008 mm; 95% CI: 0.006, 0.010). Weight at 15 years was also associated with an increase in AXL (0.005 mm; 95% CI: 0.001, 0.009).

**Table 4:** Associations between teenage factors height and weight and refractive error mean spherical equivalent (MSE dioptres), axial length (AXL mm) and radius of corneal curvature (RCC mm) measured at age 15 years

	Multivariable analysis	***P*** value
**Mean Spherical Equivalent**	**(ß coefficient (95% CI))**	
Teenage Weight (kg)	0.002 (− 0.0015,0.006)	*p* = 0.254
Teenage Height (cm)	−0.007 (− 0.013,-0.001)	*p* = 0.028
**Axial length**		
Teenage Weight (kg)	0.005 (0.001,0.009)	*p* = 0.023
Teenage Height (cm)	0.021 (0.015,0.028)	p < 0.001
**Corneal Curvature**		
Teenage Weight (kg)	0.0002 (−0.001,0.001)	*p* = 0.730
Teenage Height (cm)	0.008 (0.006,0.010)	p < 0.001

## Discussion

The anthropometric measures of height and weight, but not BMI, across the life-course demonstrate an association with the optical biometric measures of AXL and RCC measured at age 15 years. However the negative association between refractive error (MSE) and an increase in height was only found to be associated at the teenage life stage (age 15 years) and no other life stage.

BMI is commonly used as a measure of obesity in adults however in children and adolescents there is no agreed definition of BMI relating to health outcomes, this is due to greater variation in body fat content in comparison to adults [12, 13]. Height and weight are therefore believed to be more accurate predictors of obesity in children and adolescents [13]. This may contribute to our finding that height and weight, but not BMI demonstrated an association with ocular structure.

The association we report of increasing maternal height with increasing offspring AXL and RCC is comparable to previous reporting of a relationship between height and ocular dimensions [14], we additionally report an association between increase in maternal pre-natal weight and offspring AXL at age 15 years. The positive relationship we report between longer and heavier babies and increased AXL and larger RCC has been shown previously [15] with this positive relationship reported during early childhood [3] however our findings indicate that these relationships are consistent and continue beyond the onset of puberty. The associations we report between maternal height, birth length and height at age 15 years may be due to a combination of shared genetic and environmental factors. Maternal lifestyle is known to be strongly associated with both improved nutrition and increased height in childhood, with older better educated mothers reported to influence the child’s environment positively, and hence improve their child’s development [16].

The lack of association of MSE in the prenatal and birth models we report is similar to findings in a study of Singapore Chinese children who demonstrated an association between AXL and birth length and weight but not refractive error at age 7–9 years [17]. Refractive change occurs throughout the life-course with greater levels of hyperopia in young children followed by the process of emmetropisation which can lead to the potential development of myopia [6]. Myopia prevalence increases with age in Western populations and is rarely found in young children, it is commonly referred to as “school myopia” with increased incidence around the time of puberty, a time of increased growth [18].

Previous studies reporting the relationship between refractive error, height and weight have reported varying results, with Rosner et al. finding no association between myopia and stature in conscripted male teenagers [19] and Rahi et al. reporting a relationship between myopia and height in an adult population at age 44 years [5]. Our study found AXL and RCC associated with height and weight across the life course analyses but a decrease in MSE (increase in myopia) was only found to be associated with an increase in height at age 15 years. The variance between studies reporting the association of height with MSE, AXL and RCC may be influenced in part by the age at which the measures were collected with growth patterns and myopic refractive error being influenced by puberty [18]. The female children in this study are known to have progressed through puberty by age 15 years [20] and this may have influenced the change in association between height and MSE with increased height associated with an increase in myopia, reported only at the teenage life stage. Growth at puberty is complex and not fully understood, and there are many environmental factors that combine with a person’s genetic susceptibility to influence growth and eventual height [21, 22]. When energy stores are suitable for puberty, biochemical reactions take place in the arcuate nucleus of the hypothalamus with the hormone leptin playing a role in pubertal development stimulating the pulsatile release of gonadotrophin-releasing hormone (GnRH) [23]. Increase in adiposity at the point of puberty triggers the release of leptin and in order to maintain hormonal balance, the suppression of dopamine occurs [24].

A study of the allelic score from 180 SNPs associated with adult height has been undertaken for this ALSPAC cohort with children having an above average increase in the height allelic score having a flatter corneal curvature [2]. A GWAS study of refractive error [8] which reported an overlap of genes with anthropometric traits also presented a number of newly identified genes related to the physiological process of light stimulation and visual information processing; in particular a genetic association of the DRD1 gene which is linked to the dopaminergic pathway is reported.

Dopamine has been suggested as a possible hormonal mechanism influencing the development of myopia with lower levels of dopamine and increased axial length reported in low light levels [25]. There are many studies in the field of growth and development presenting the regulatory mechanism occurring between leptin and dopamine [24, 26, 27] and there are a number of studies reporting the influence of dopamine on axial length growth in myopia [25, 28] but the authors are not aware of studies reporting interaction between leptin and dopamine in the development of myopia. This may be an important area that may benefit from further investigation.

Our data indicates an association between anthropomorphic measures, ocular biometry and refractive error and supports the theory that refractive error results from a complex interaction of genetic and environmental factors [8], adding to our understanding of the development of myopia and informing future clinical treatment. The strengths of this study are the large sample size with measures at key life stages; pre-natal, birth and teenage. The inclusion of teen measurements provides the opportunity to explore the influence of puberty, a period of significant growth. There are also a number of limitations. The measure of refractive error (MSE) was obtained from non-cycloplegic autorefraction, and this is likely to have over-estimated the number of myopic children. An independent evaluation of the MSE indicates a small (− 0.25D) systematic bias [29]; however, this would not influence the reported relationships and neither should it affect the AXL or RCC results. The onset of myopia is reported at a younger age in East Asian populations and we do not include a measure of ethnicity as the cohort is predominately of white British origin, our findings will therefore not be representative of other more ethnically diverse populations. We were not able to adjust for all potential confounders in our models, measures for time spent outdoors with an accelerometer previously reported in the ALSPAC children was not used in these analyses due to the small sample available [30]. No measure of sustained close work or childhood educational attainment were included, although all children were in the same age group and had the same number of school years in mandatory education.

## Conclusion

Our findings are consistent with a small number of studies reporting association between anthropomorphic measures and ocular parameters [3, 5, 17] and add to the understanding of ocular structure, refractive error and myopia and their changing relationship with height and weight. Further research is required into the development of refractive error over the life-course, the hormonal mechanism for general growth particularly at the time of puberty and how this impacts on refractive growth patterns along with the mediating effect of environmental factors, including maternal lifestyle, on refractive development at each life stage.

## Data Availability

Please note that the ALSPAC study website contains details of all the data that is available through a fully searchable data dictionary and variable search tool. http://www.bristol.ac.uk/alspac/researchers/our-data/.

## Abbreviations

ALSPAC: Avon Longitudinal Study of Parents and Children
AX: Axial length
BMI: Body Mass Index
CI: Confidence Interval
GWAS: Genome-wide association meta-analysis
GnRH: Gonadotrophin-releasing hormone
MSE: Mean spherical equivalent
RCC: Radius of corneal curvature
SNP’s: Single-nucleotide polymorphism
SD: Standard Deviation

## References

[1] Barker DJ (2004). The developmental origins of chronic adult disease. Acta Paediatr Suppl.

[2] Northstone K, Guggenheim JA, Howe LD (2013). Body stature growth trajectories during childhood and the development of myopia. Ophthalmology.

[3] Tideman JWL, Polling JR, Jaddoe VWV, Vingerling JR, Klaver CCW (2019). Growth in foetal life, infancy, and early childhood and the association with ocular biometry. Ophthalmic Physiol Opt.

[4] Freeman DJ (2010). Effects of maternal obesity on fetal growth and body composition: implications for programming and future health. Semin Fetal Neonatal Med.

[5] Rahi JS, Cumberland PM, Peckham CS (2011). Myopia over the lifecourse: prevalence and early life influences in the 1958 British birth cohort. Ophthalmology.

[6] Mutti DO, Hayes JR, Mitchell GL (2007). Refractive error, axial length, and relative peripheral refractive error before and after the onset of myopia. Invest Ophthalmol Vis Sci.

[7] Meng W, Butterworth J, Malecaze F (2011). Axial length of myopia: a review of current research. Ophthalmologica.

[8] Tedja MS, Wojciechowski R, Hysi PG (2018). Genome-wide association meta-analysis highlights light-induced signaling as a driver for refractive error. Nat Genet.

[9] Fraser A, Macdonald-Wallis C, Tilling K (2013). Cohort profile: the Avon longitudinal study of parents and children: ALSPAC mothers cohort. Int J Epidemiol.

[10] Boyd A, Golding J, Macleod J (2013). Cohort profile: the ‘children of the 90s’--the index offspring of the Avon longitudinal study of parents and children. Int J Epidemiol.

[11] Ouyang L-J, Yin Z-Q, Ke N (2015). Refractive status and optical components of premature babies with or without retinopathy of prematurity at 3-4 years old. Int J Clin Exp Med.

[12] Pietrobelli A, Faith MS, Allison DB (1998). Body mass index as a measure of adiposity among children and adolescents: a validation study. J Pediatr.

[13] Savva SC, Tornaritis M, Savva ME (2000). Waist circumference and waist-to-height ratio are better predictors of cardiovascular disease risk factors in children than body mass index. Int J Obes Relat Metab Disord.

[14] Nangia V, Jonas JB, Matin A (2010). Body height and ocular dimensions in the adult population in rural Central India. The Central India eye and medical study. Graefes Arch Clin Exp Ophthalmol.

[15] Friling R, Weinberger D, Kremer I, Avisar R, Sirota L, Snir M (2004). Keratometry measurements in preterm and full term newborn infants. Br J Ophthalmol.

[16] Jackson MI, Kiernan K, McLanahan S (2017). Maternal education, changing family circumstances, and children’s skill development in the United States and UK. Ann Am Acad Pol Soc Sci.

[17] Saw SM, Tong L, Chia KS (2004). The relation between birth size and the results of refractive error and biometry measurements in children. Br J Ophthalmol.

[18] Yip VC, Pan CW, Lin XY (2012). The relationship between growth spurts and myopia in Singapore children. Invest Ophthalmol Vis Sci.

[19] Rosner M, Laor A, Belkin M (1995). Myopia and stature: findings in a population of 106,926 males. Eur J Ophthalmol.

[20] Christensen KY, Maisonet M, Rubin C (2010). Pubertal pathways in girls enrolled in a contemporary British cohort. Int J Pediatr.

[21] Burt Solorzano CM, McCartney CR (2010). Obesity and the pubertal transition in girls and boys. Reproduction.

[22] Catalano PM, Ehrenberg HM (2006). The short- and long-term implications of maternal obesity on the mother and her offspring. BJOG.

[23] Shalitin S, Phillip M (2003). Role of obesity and leptin in the pubertal process and pubertal growth--a review. Int J Obes Relat Metab Disord.

[24] Beeler JA, Faust RP, Turkson S (2016). Low dopamine D2 receptor increases vulnerability to obesity via reduced physical activity, not increased appetitive motivation. Biol Psychiatry.

[25] Feldkaemper M, Schaeffel F (2013). An updated view on the role of dopamine in myopia. Exp Eye Res.

[26] Hommel JD, Trinko R, Sears RM (2006). Leptin receptor signaling in midbrain dopamine neurons regulates feeding. Neuron.

[27] Belcher BR, Chou CP, Nguyen-Rodriguez ST (2013). Leptin predicts a decline in moderate to vigorous physical activity in minority female children at risk for obesity. Pediatr Obes.

[28] Weiss S, Schaeffel F (1993). Diurnal growth rhythms in the chicken eye: relation to myopia development and retinal dopamine levels. J Comp Physiol A.

[29] Williams C, Miller L, Northstone K (2008). The use of non-cycloplegic autorefraction data in general studies of children’s development. Br J Ophthalmol.

[30] Guggenheim JA, Northstone K, McMahon G (2012). Time outdoors and physical activity as predictors of incident myopia in childhood: a prospective cohort study. Invest Ophthalmol Vis Sci.

